# Echocardiographic guidance in transcatheter tricuspid valve interventions

**DOI:** 10.1186/s44348-026-00079-4

**Published:** 2026-07-15

**Authors:** Kevin Ka Ho Kam, Mani Vannan, Alex Pui Wai Lee

**Affiliations:** 1 Division of Cardiology, Department of Medicine and Therapeutics, Prince of Wales Hospital, Hong Kong SAR, China; 2 Marcus Heart Valve Center, Piedmont Heart Institute, Atlanta, GA USA; 3 Li Ka Shing Institutes of Health Science, Prince of Wales Hospital, The Chinese University of Hong Kong, Hong Kong SAR, China

**Keywords:** Transesophageal echocardiography, Transcatheter tricuspid valve interventions, Transcatheter tricuspid edge-to-edge repair, Transcatheter tricuspid valve replacement

## Abstract

Echocardiographic guidance plays a pivotal role in pre-procedural screening and intraprocedural guidance for transcatheter tricuspid valve interventions (TTVI). Pre-procedurally, comprehensive transesophageal echocardiography (TEE) is essential to understand the tricuspid regurgitation (TR) mechanism, quantify TR severity, tricuspid leaflet morphology, right ventricular function/size, and help determine transcatheter tricuspid edge-to-edge repair (T-TEER) and transcatheter tricuspid valve replacement (TTVR) selection based on underlying tricuspid valve anatomy. Interprocedurally, the use of real-time three-dimensional transesophageal echocardiography (3D-TEE) together with fluoroscopy allows precise guidance of device steering, leaflet insertion, and deployment while minimizing complications, especially in technically challenging tricuspid valve anatomy. Post-device deployment echocardiography assists in the assessment of residual TR, paravalvular leak, transvalvular gradient, right ventricular function changes, and the occurrence of pericardial effusion. As device technologies and indications for TTVI continue to expand, high-quality echocardiographic protocols and expertise are central to optimizing patient selection, procedural safety, and long-term outcomes in transcatheter treatment of tricuspid valve regurgitation.

## Background

Tricuspid regurgitation (TR) has emerged as a global health challenge and is strongly associated with adverse cardiovascular outcomes [1]. If left untreated, TR leads to progressive right heart failure [2]. Isolated surgical tricuspid valve (TV) intervention is infrequently performed [3] and is associated with an in-hospital mortality rate of approximately 10% [4]. This has driven the development of less invasive transcatheter solutions, which are rapidly transforming the therapeutic paradigm. According to the European Society of Cardiology guidelines, transcatheter TV intervention is recommended as class IIa indication [5]. It should be considered to improve quality of life and promote right ventricular (RV) remodeling in high-risk patients with symptomatic severe TR despite optimal medical therapy, provided that severe RV dysfunction or precapillary pulmonary hypertension is absent. Both transcatheter tricuspid edge-to-edge repair (T-TEER) and transcatheter TV replacement (TTVR) have demonstrated safety and efficacy in reducing TR severity and are being increasingly adopted worldwide [6, 7]. This study primarily focuses on the intraprocedural echocardiographic imaging required for T-TEER (with TriClip [Abbott Laboratories])) and TTVR (with LuX-Valve Plus [Ningbo Jenscare Biotechnology] and EVOQUE [Edwards Lifesciences]).

## Baseline preprocedural echocardiographic imaging and medication optimization

It is difficult to fully study the TV anatomy based on transthoracic echocardiography alone. Therefore, comprehensive baseline transesophageal echocardiography (TEE) is a prerequisite for all patients with significant TR being considered for transcatheter tricuspid valve intervention (TTVI), i.e., T-TEER or TTVR. It delineates TR severity, etiology, jet location and width, coaptation gap, TV annular dimensions, and leaflet anatomy and morphology [9]. Optimization of diuretics are recommended (e.g., loop diuretics, mineralocorticoid receptor antagonists) prior to TEE to optimize the volume status of every patient with significant TR [5]. Suitable patients should be put on sodium-glucose cotransporter 2 inhibitors. This imaging provides the heart team with essential parameters to decide the optimal treatment strategy (surgery, transcatheter intervention, or medical therapy) and helps select the appropriate TTVI device [10].

### TEE imaging protocol

Imaging the tricuspid valve with TEE is technically challenging because the TV is the most anterior and inferiorly located native valve, with an orifice area of up to 7 to 9 cm^2^ [11], which requires a wide field of view. Its oblique orientation relative to the esophagus makes it impossible to align the annulus perpendicularly (90°) to ultrasound scan lines, increasing reliance on lateral resolution [9]. Furthermore, left heart structures (e.g., aortic or mitral valve prostheses, thickened interatrial septum, atrial septal occluder devices) can cause significant shielding artifacts at the mid-esophageal (ME) level. Therefore, deep esophageal and transgastric (TG) views provide indispensable imaging guidance for both preprocedural screening and during the TTVI procedure.

### TR severity grading

**Fig. 1:**
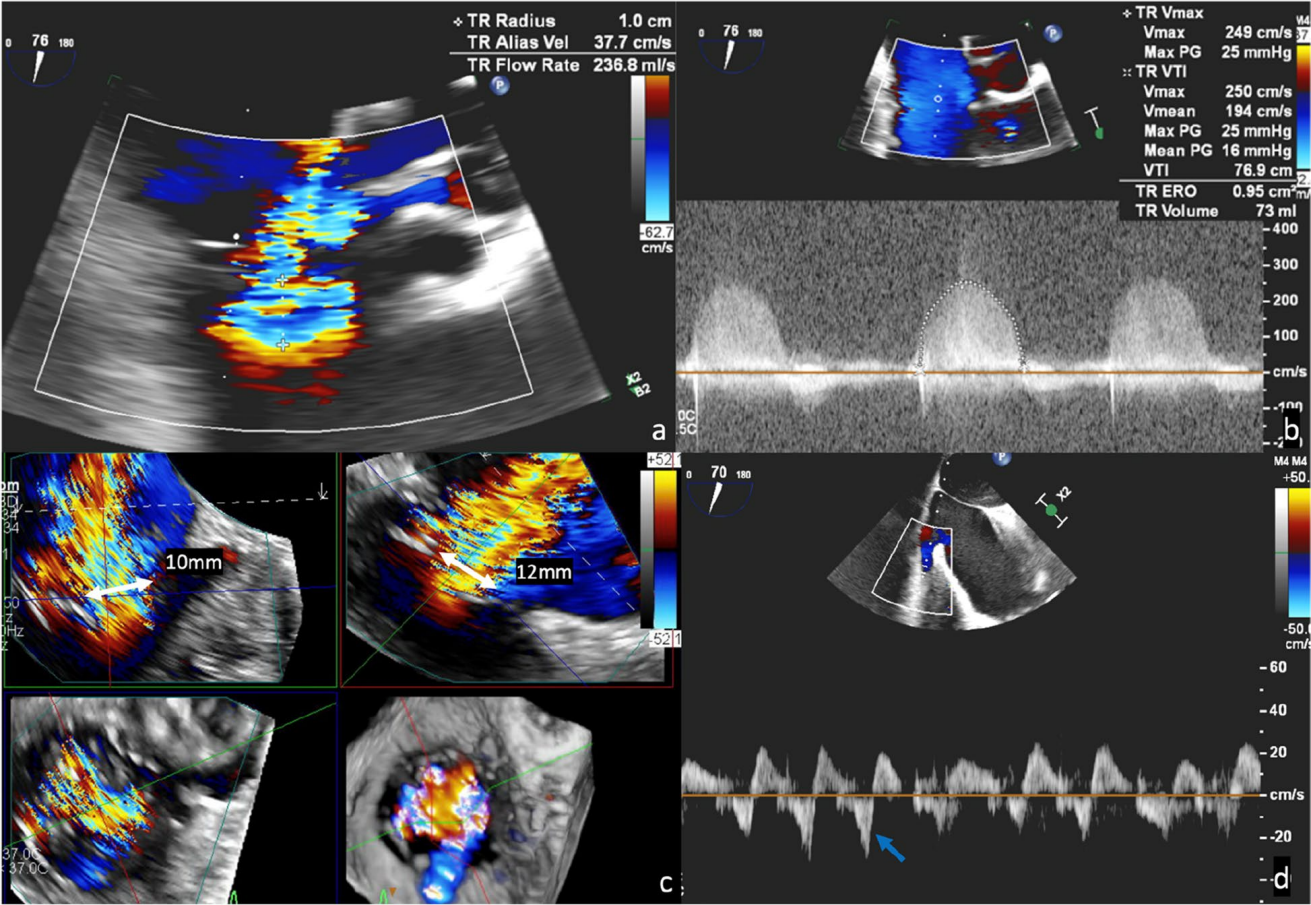
Multiple parametric methods to determine severity of tricuspid regurgitation (TR). **A**, **B** Torrential TR as demonstrated by 2D effective regurgitant orifice area (EROA) of 0.95 cm^2^ and TR regurgitant volume of 73 mL which are derived from proximal isovelocity surface area (PISA) method and TR signal tracing. **C** Evidence of slightly asymmetric vena contracta diameters (10 and 12 mm; double-headed white arrows) in 3D multiplanar reconstruction imaging, which is consistent with severe TR (average vena contracta diameter of 11 mm). **D** Presence of systolic flow reversal (blue arrow) from hepatic vein in every cardiac cycles is a specific sign of significant TR

The latest proposed classification for TR uses a five-grade scale: mild, moderate, severe, massive, and torrential, established through a combination of parameters [12]. Massive and torrential TR are associated with a higher risk of procedural failure and significant residual TR after T-TEER [13]. Applying multiple parametric methods to determine severity is recommended to improve overall specificity [9]. Figure 1A and B demonstrate torrential TR, with a 2D effective regurgitant orifice area (EROA) of 0.95 cm^2^ and a regurgitant volume of 73 mL derived from the proximal isovelocity surface area (PISA) method. Figure 1C shows a slightly asymmetric vena contracta width with an average of 11 mm in 3D multiplanar reconstruction (MPR) imaging, consistent with severe TR. The presence of systolic flow reversal in the hepatic vein in every cardiac cycle is a specific sign of significant TR, as depicted in Fig. 1D.

### Etiologies and functional classification of TR

The most common cause of TR is secondary (functional) TR, typically resulting from RV dilation and dysfunction due to left-sided heart disease or pulmonary hypertension. A prospective cohort study demonstrated that 93% of cases are secondary TR, with the remainder being primary [14]. Among primary TR, the most frequent causes are cardiac implantable electronic device (CIED)-related and TV prolapse. Other causes include rheumatic disease, endocarditis, carcinoid disease, or traumatic injury [15]. Secondary TR is associated with the following: (1) left-sided heart disease (e.g., mitral/aortic valve disease, left ventricular dysfunction); (2) pulmonary hypertension (precapillary or postcapillary); (3) RV systolic dysfunction or dilatation; and (4) right atrium (RA) dilatation secondary to chronic atrial fibrillation.

TR can also be classified by leaflet motion according to the Carpentier functional classification [15]:

- Class I: Normal leaflet motion with severe annular dilatation (e.g., atrial functional TR [AFTR]).
- Class II: Excessive leaflet motion (e.g., prolapse).
- Class IIIa: Restricted leaflet motion in systole and diastole (e.g., rheumatic TR).
- Class IIIb: Systolic leaflet tethering due to RV dilatation and papillary muscle displacement (e.g., ventricular functional TR [VFTR]).

In essence, AFTR is characterized by severe RA dilatation with preserved RV size and function and minimal leaflet tethering. VFTR is characterized by significant leaflet tethering secondary to RV dilation and/or systolic dysfunction.

## Tricuspid transcatheter edge-to-edge repair

T-TEER adopts the Alfieri stitch concept, enhancing leaflet coaptation to reduce TR [18]. The TRILUMINATE pivotal trial demonstrated that T-TEER, compared with medical therapy, reduced a composite endpoint of death from any cause or TV surgery, heart failure hospitalization, and improved quality of life (measured by the Kansas City Cardiomyopathy Questionnaire [KCCQ] score), an effect largely driven by quality-of-life improvement [6]. At 2 years, evidence emerged of a reduction in heart failure hospitalizations in the intervention group, despite substantial crossover to intervention [8]. The subsequent 3-year results showed persistent TR reduction to ≤ moderate achieved in 79% of patients [19].

In the Asia–Pacific region, T-TEER adoption has been limited by device availability and reimbursement policies [20]. Early real-world registry data from Asia demonstrated a device success rate (TR ≤ moderate at 30 days) of 74.0% and a promising safety profile, with a 30-day major adverse event rate of 1.9% [21]. This study focuses on TriClip T-TEER under TEE guidance. Table 1 illustrates anatomical feasibility for T-TEER, highlighting favorable, feasible, and unfavorable anatomies.

**Table 1:** Detailed illustration of anatomical feasibility for T-TEER procedure

Criterion for T-TEER procedure	Favorable anatomy	Feasible anatomy	Unfavorable anatomy
TR jet location	Septoanterior or central	Septoposterior	Anteroposterior
TR severity	Severe to massive	Torrential	Not applicable
Septolateral coaptation gap	≤ 7 mm	7–9 mm	≥ 10 mm
Leaflet morphology	Trileaflet	Bileaflet/quadrileaflet	Stellate morphology
TR etiology	AFTR, VFTR, localized TV prolapse	Significant TV prolapse with wide flail gap/width	Rheumatic or carcinoid disease
CIED relationship with TV	CIED-associated TR	CIED-related TR (TV leaflet impingement) with anticipated TR reduction	CIED-related TR with unlikely TR reduction

The T-TEER is proceeded in the favorable anatomgy and can be attempted in the feasible anatomy. For unfavorable anatomgy, orthotopic transcatheter tricuspid valve replacement should be considered instead of T-TEER

AFTR, atrial functional tricuspid regurgitation; CIED, cardiac implantable electronic device; TR, tricuspid regurgitation; T-TEER, tricuspid transcatheter edge-to-edge repair; TV, tricuspid valve; VFTR, ventricular functional tricuspid regurgitation

## TV leaflet assessment

**Fig. 2:**
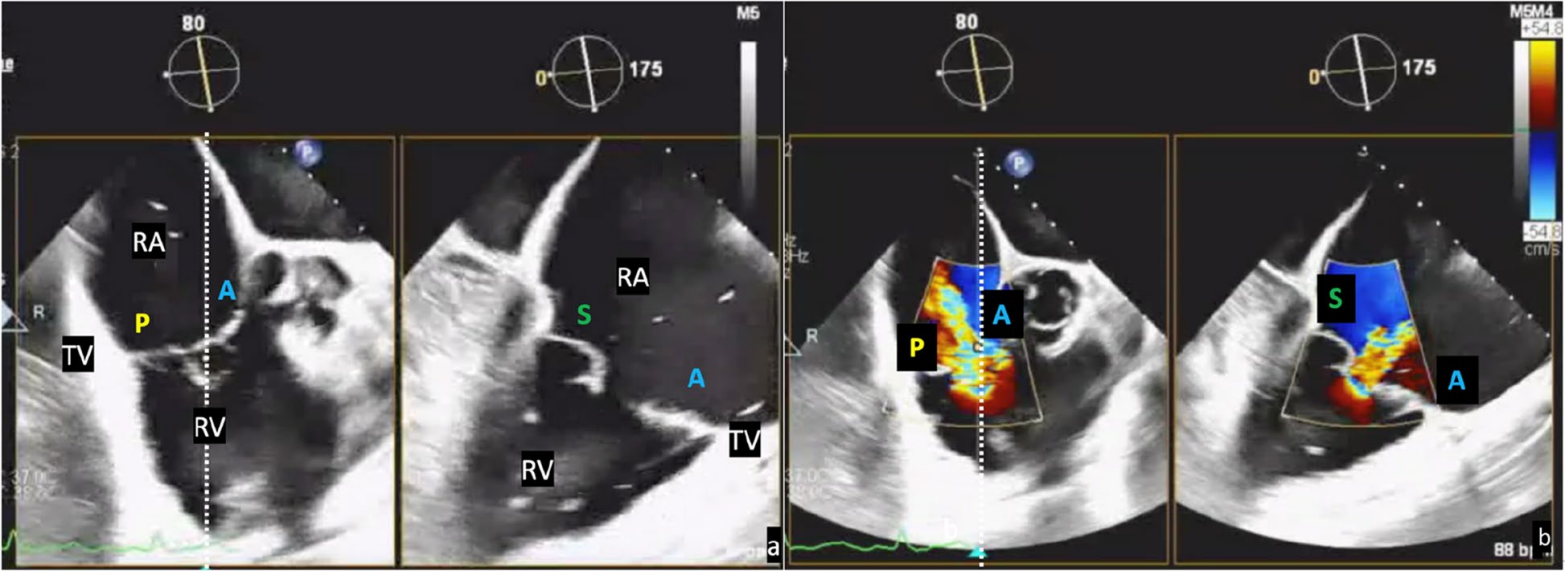
The mid-esophageal right ventricle (RV) inflow-outflow view. **A** Using simultaneous biplane imaging at RV inflow-outflow view (50°–70°), sweeping the orthogonal imaging plane (dotted line) from posterior (P; left side of primary view) to anterior (A; right side of primary view, near the aortic valve) visualizes the coaptation of the posterior-septal (P-S), and anterior-septal (A-S) commissures respectively. **B** The color flow Doppler from biplane imaging helps to localize the tricuspid regurgitation (TR) jet, which is more significant in central and anteroseptal aspect. RA, right atrium; RV, right ventricle; TV, tricuspid valve

The ME RV inflow-outflow view (50°–70°) visualizes the TV anterior and posterior leaflets [23]. Using biplane imaging of the ME RV inflow-outflow view (Fig. 2), the orthogonal view will be the ME grasping view; sweeping the biplane cursor on ME RV inflow-outflow view from posterior to anterior leaflets visualizes the coaptation of the posterior-septal and anterior-septal commissures; this allows assessment of anterior and posterior leaflet length, chordal involvement, and coaptation gaps.

**Fig. 3:**
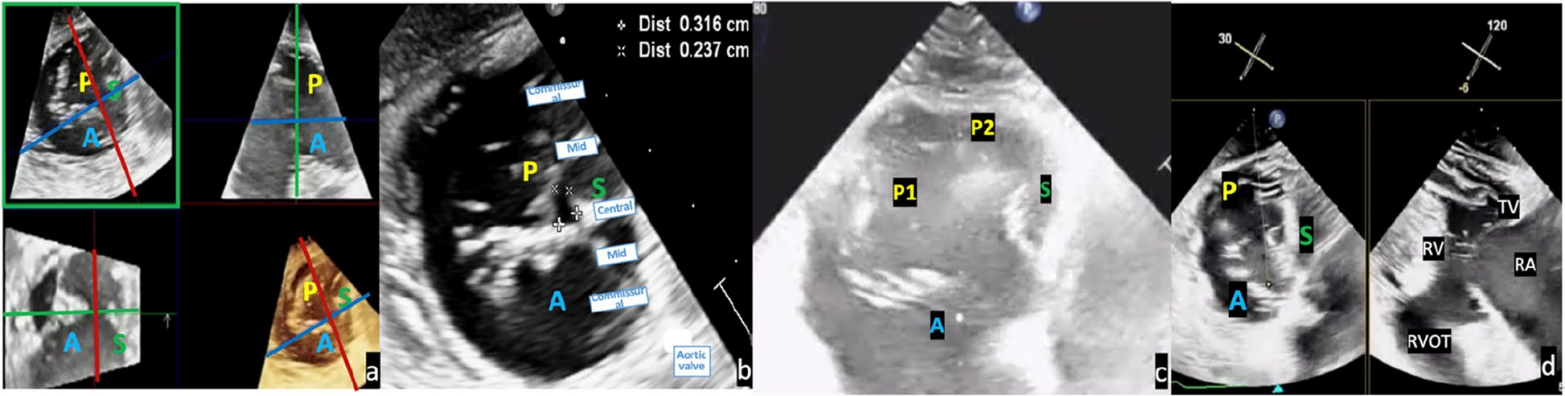
Tricuspid valve (TV) leaflet anatomy in transgastric (TG) short-axis view (0°–30°). **A** The 3D multiplanar reconstruction imaging at this level allows in-depth assessment of number of leaflets (anterior leaflet, A; posterior leaflet, P; septal leaflet, S), leaflet morphology (thickness, calcification), chordal attachment, and pacing/defibrillator lead involvement if any. The red plane is cutting through A-P leaflets; the blue plane is cutting through A-S leaflets; the green plane is the *en face* view of TV. **B** The TV short-axis view with aortic valve at 5 o’clock is essential to identify the maximum coaptation gap, which is localized at central orifice measured 3.2 mm. **C** The TV anatomy in TG short-axis view (0°–30°) showing a four-leaflet TV with anterior (A) and septal (S) leaflets, and two posterior leaflets (P1 and P2). **D** The TV imaging from TG view (30°) is guided and further optimized by using two-chamber inflow-outflow view of right heart (120°). RA, right atrium; RV, right ventricle; RVOT, right ventricular outflow tract

The 2D (0°–30°) or 3D TG view focusing on the short-axis view of the TV helps identify the number of leaflets, TR localization, chordal attachment, and maximum coaptation gaps (Fig. 3A, B). A coaptation gap ≥ 10 mm is independently associated with higher cardiovascular mortality [16]. The TG view is optimal for visualizing leaflet number; a ≥ 4-leaflet morphology is considered challenging for T-TEER (Fig. 3C) [17]. The TV imaging from TG view (30°) can be further optimized by using two-chamber inflow-outflow view of right heart (120°) in the situation of challenging TG view (Fig. 3D). The normal transtricuspid gradient, assessed by continuous-wave Doppler, is typically 0 to 2 mmHg. TV area can be estimated by 3D MPR with the cut plane aligned at the leaflet tips in mid-diastole.

## Intraprocedural guidance of T-TEER with echocardiography

**Fig. 4:**
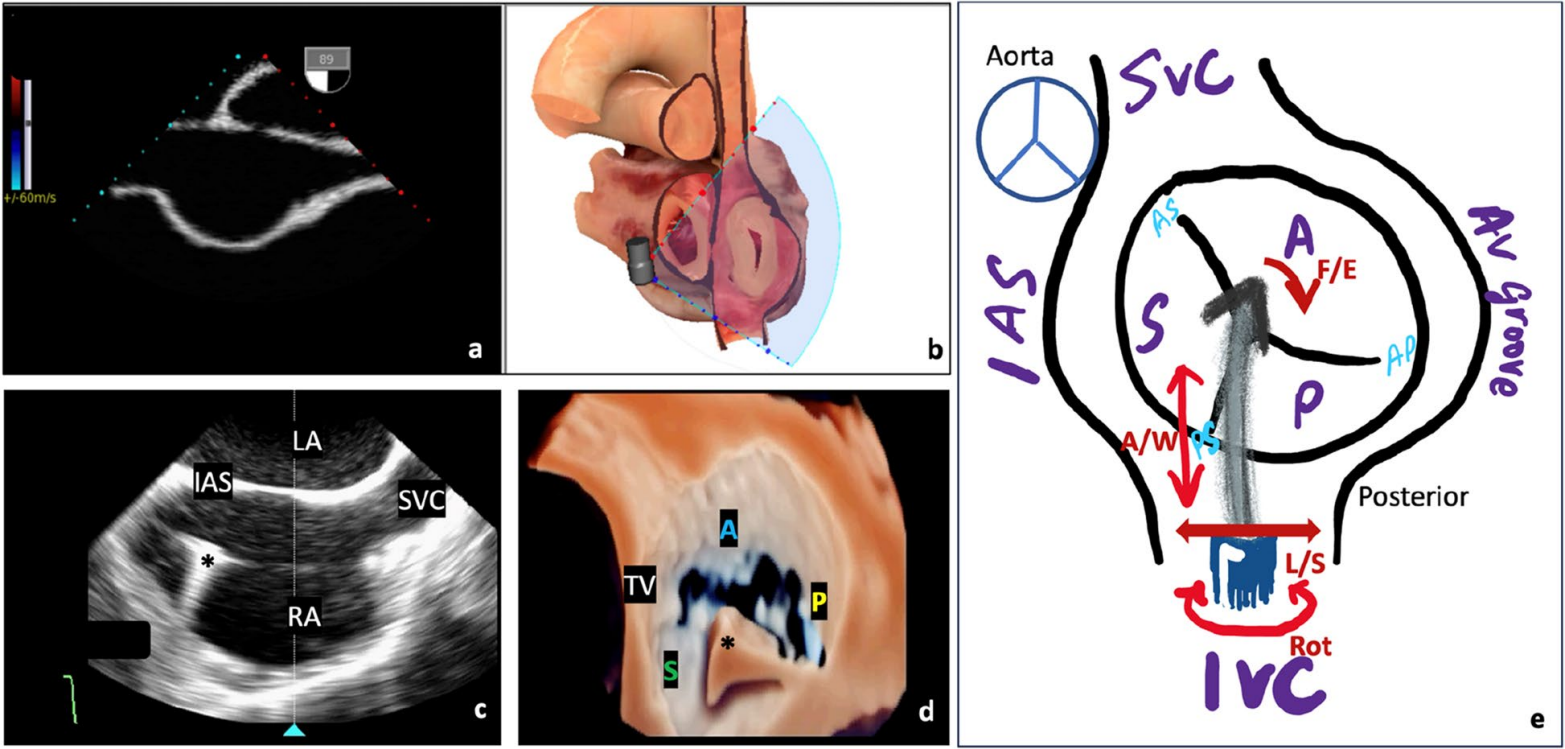
Echocardiographic and schematic visualization of the TriClip system (Abbott Laboratories) for tricuspid valve (TV) edge-to-edge repair. **A** Simulated transesophageal echocardiogram (TEE) image of a standard bicaval view. **B** Simulated cardiac structures adjacent to TEE probe, highlighting its anatomical relationship to the TV. **C** Intraprocedural TEE image (bicaval view) with the TriClip clip delivery system (asterisk) positioned. **D** The 3D TEE image of the bicaval view, rotated 90° counterclockwise to profile the TV and TriClip delivery system (asterisk). **E** Schematic diagram illustrating the primary steering motions of the TriClip system: advancement/withdrawal (A/W), catheter rotation (Rot), flexion/extension (F/E), and lateral/septal (L/S) control. A, anterior leaflet; AP, anteroposterior commissure; AS, anteroseptal commissure; AV, aortic valve; IAS, interatrial septum; IVC, inferior vena cava; LA, left atrium; P, posterior leaflet; PS, posteroseptal commissure; RA, right atrium; S, septal leaflet; SVC, superior vena cava

The procedural workflow begins with advancement of the guide catheter into the RA via the inferior vena cava. Under the bicaval view (90°–110°), the clip delivery system (CDS) is navigated into the RA. To subsequently maneuver and flex the CDS toward the tricuspid annulus, the 3D *en face* view of the TV is recommended (Fig. 4A–D) while avoiding contact with the atrial septum [15]. Initial CDS movements follow a predictable mechanical principle (Fig. 4E).

**Fig. 5:**
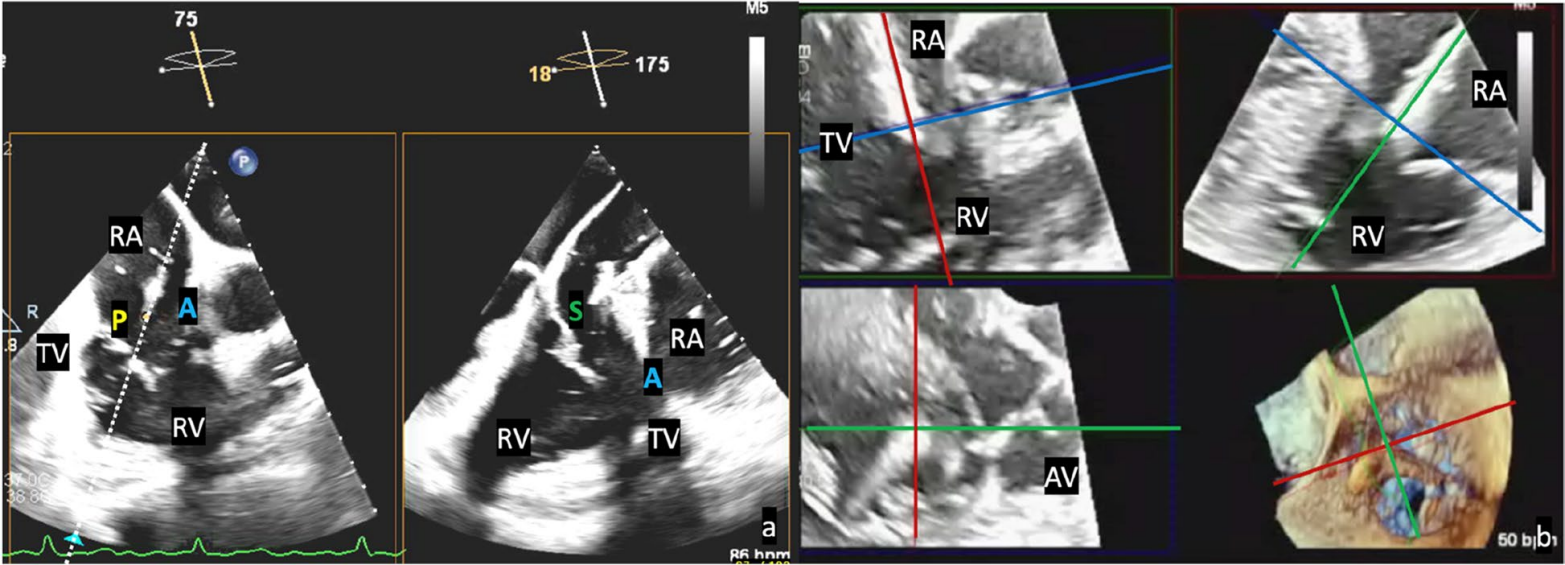
**A** Once the device delivery system has approached just above the tricuspid valve plane, the right ventricular (RV) inflow-outflow mid-esophageal (ME) view (50°–70°) and orthogonal four-chamber view (150°–180°) assists in directing device trajectory and location. **B** The optimal trajectory pathway should be perpendicular to the tricuspid annular plane which could be well demonstrated from 3D multiplanar reconstruction of RV inflow-outflow ME view (with embedded video). A, anterior leaflet; AV, aortic valve; P, posterior leaflet; RA, right atrium; S, septal leaflet; TV, tricuspid valve

Once positioned above the TV, the device trajectory and location are optimized using the ME RV inflow-outflow view paired with an orthogonal leaflet grasping view (approximately 150°–180°) (Fig. 5A). The ideal trajectory is orthogonal to the tricuspid annular plane, best visualized with 3D MPR imaging (Fig. 5B).

**Fig. 6:**
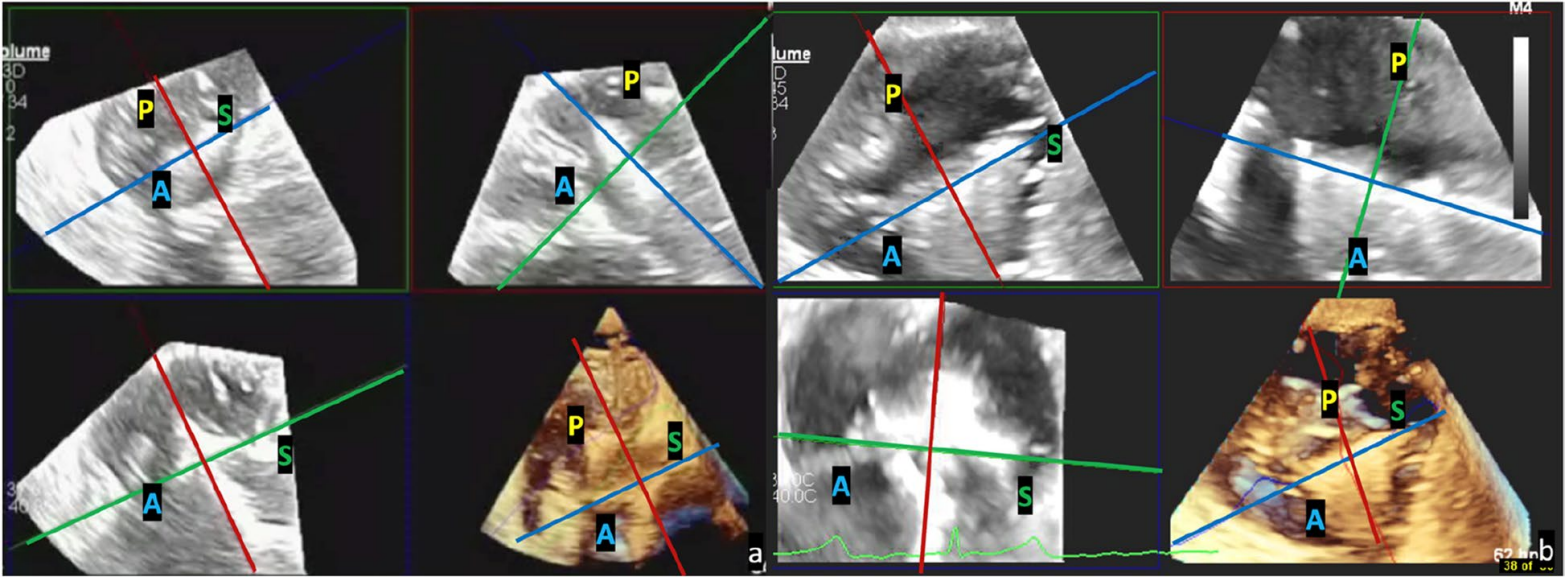
The transgastric (TG) 3D multiplanar reconstruction (MPR) views. **A** The TG 3D MPR view (0°–30°) focusing on tricuspid valve short-axis level provides pivotal information on clip arm orientation, in which the clip arm is rotated to 2–8 o’clock orientation (with embedded video). **B** Once the clip delivery system is 2 to 3 mm below the tricuspid valve, the clip arms would be opened and clip arm orientation has to finely adjust using TG 3D MPR view. A, anterior leaflet; P, posterior leaflet; S, septal leaflet

After confirming the trajectory, the clip arms are opened and rotated perpendicular to the coaptation line. The 2D or 3D TG short-axis views are essential for verifying clip arm orientation (Fig. 6A). The independent grasping function should be tested before advancing the device into the RV.

The CDS is closed to an angle of 60° to 120° before crossing the TV leaflets; for subsequent clips, it is typically closed completely. Once below the leaflets in the RV, the clip arms are reopened, and fine-tuning of orientation is performed using the 2D or 3D TG short-axis view (Fig. 6B). The device is then retracted toward the leaflets.

Although various implant strategies exist, the first clip is typically deployed at the site of the widest regurgitant jet, often between the anteroseptal leaflets. Given the large TV orifice and adequate leaflet length, the longer/wider TriClip XTW device is frequently selected. For particularly wide central gaps (> 7 mm), a “zip and clip” technique may be employed, where the initial clip is placed more commissurally to reduce the residual gap.

**Fig. 7:**
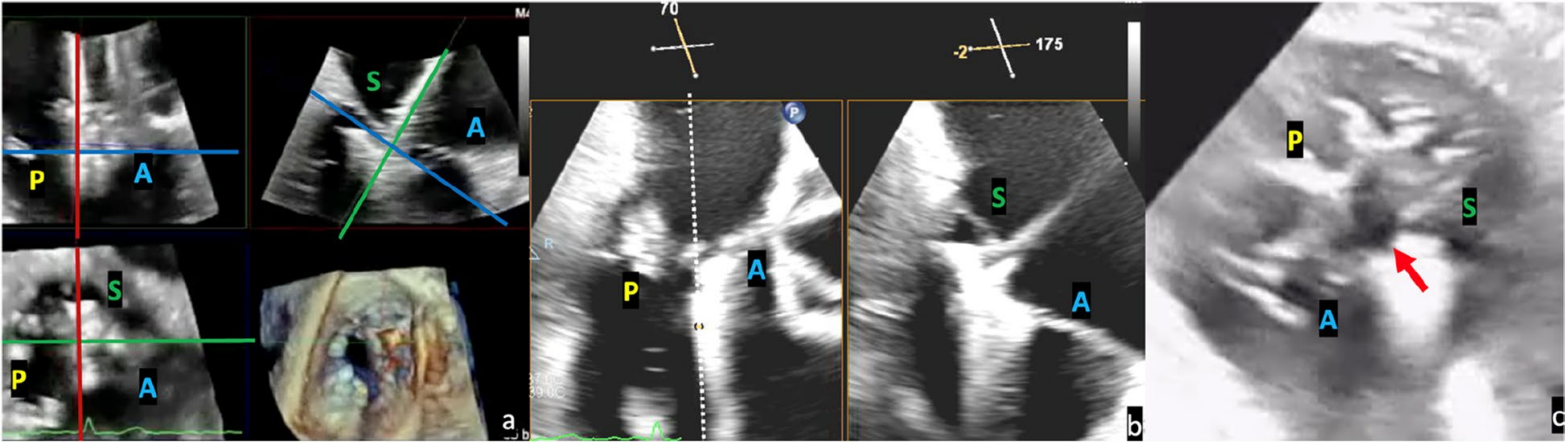
**A** The 3D multiplanar reconstruction or **B** biplane mid-esophageal right ventricle inflow-outflow view (50°–70°) provides adequate imaging guidance for leaflet capture. **C** The clips are gradually closed and make sure the tissue bridge (arrow) has been well established from transgastric short axis view of TV (with embedded video). A, anterior leaflet; P, posterior leaflet; S, septal leaflet

During leaflet capture, the TG 3D MPR view provides critical visualization. Operators should simultaneously monitor the short-axis view (assessing leaflet orientation and position; immobilization as signs of successful grasp) and the leaflet grasping plane (demonstrating leaflet insertion). Successful leaflet insertion can be confirmed in either 2D or 3D TG short-axis views, and is further guided by the ME 3D MPR or biplane ME RV inflow-outflow view (Fig. 7A, B).

**Fig. 8:**
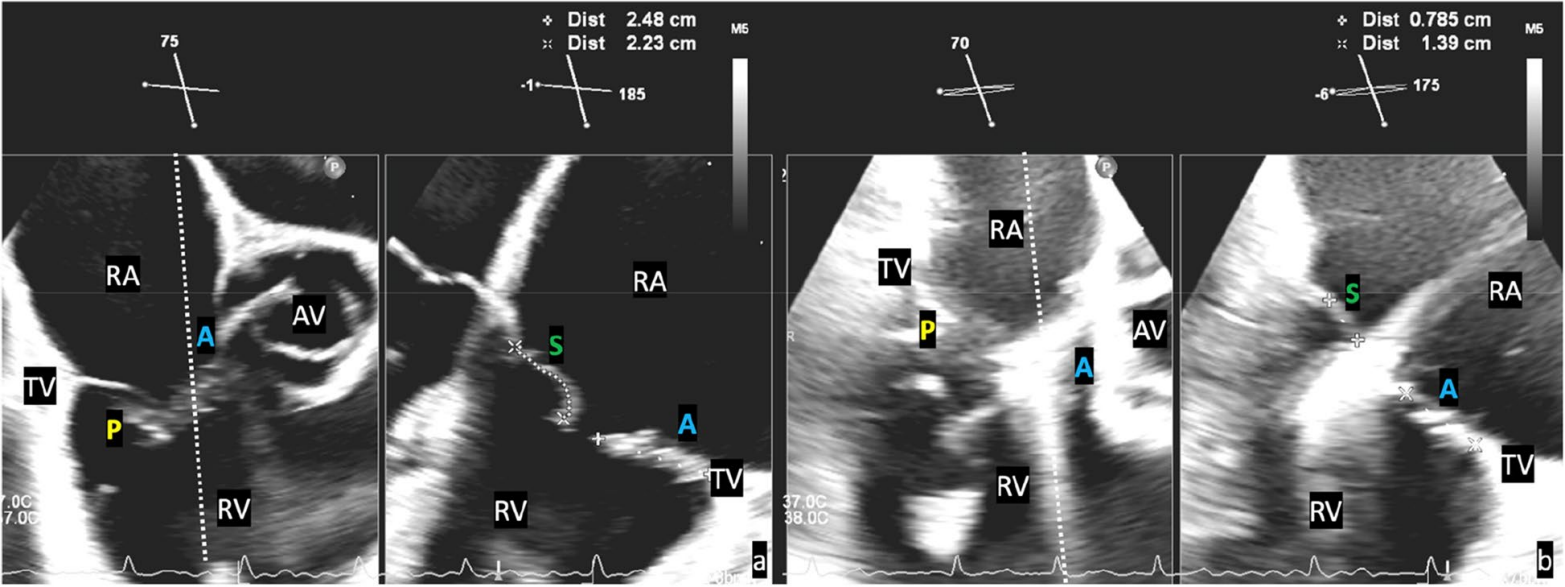
The tricuspid valve (TV) leaflet lengths at (**A**) baseline and (**B**) after clipping status should be compared and leaflet insertion should be calculated. For TriClip XTW system, the recommended leaflet lengths should be at least 9 mm or above. The septal leaflet insertion is 14 mm and anterior leaflet insertion is 11 mm, which are adequate for secure grasping. A, anterior leaflet; AV, aortic valve; P, posterior leaflet; RA, right atrium; RV, right ventricle; S, septal leaflet

Following anticipated adequate leaflet tissue insertion, the grippers are lowered. A positive “bouncing sign” indicates secure leaflet capture between the clip arms and grippers. The clip is gradually closed, and the formation of a tissue bridge should be confirmed from the TG view (Fig. 7c). Pre- and post-clip leaflet lengths should be compared. For the TriClip XT/XTW devices, the company recommends a minimum leaflet length of 9 mm (Fig. 8), whereas for the NT/NTW devices, a minimum length of 6 mm is required. Of note, one study has suggested that leaflet insertion should be at least 5 mm on both leaflets [24].

**Fig. 9:**
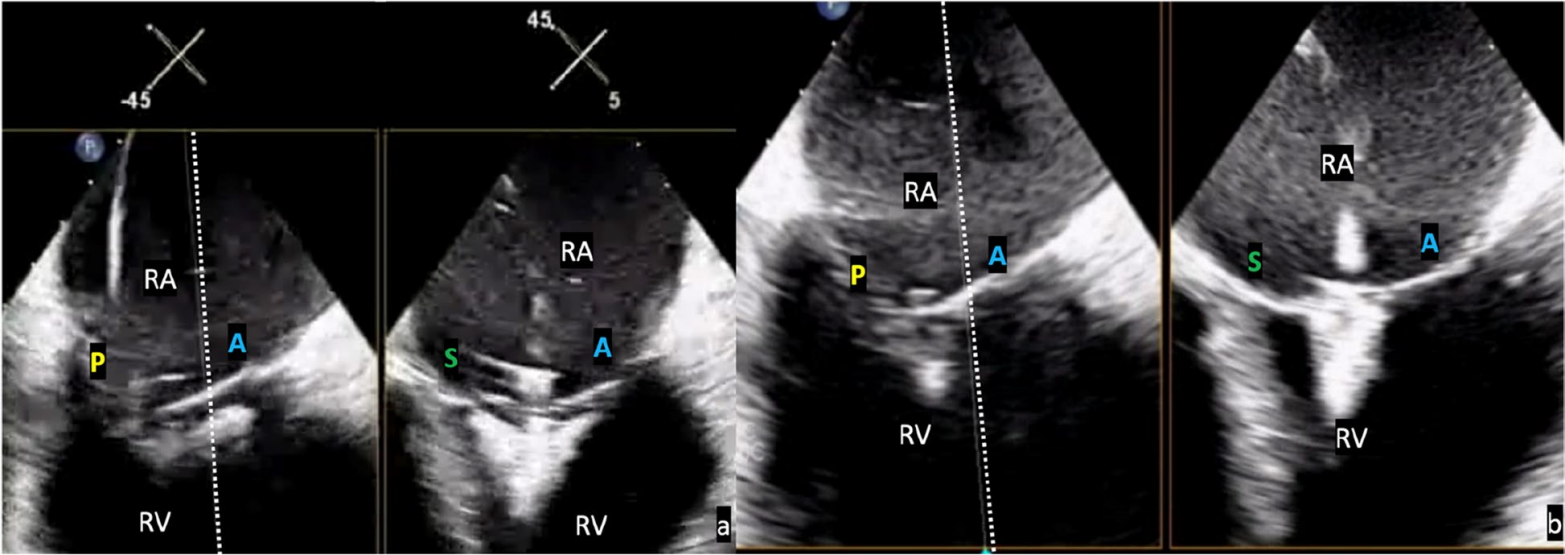
The 3D intracardiac echocardiography imaging can serve as important tool when the transesophageal echocardiography imaging is suboptimal to guide tricuspid valve (TV) leaflet grasping. The use of –45°/+ 45° view together with a left flexion could help to get the adequate images for anteroseptal leaflet grasping in this situation. **A** TV leaflet capture by TriClip. **B** Adequate TV leaflet capture. A, anterior leaflet; P, posterior leaflet; RA, right atrium; RV, right ventricle; S, septal leaflet

In cases where TEE provides suboptimal views for leaflet grasping (e.g., due to prosthetic valve or device artifact), 3D intracardiac echocardiography (ICE) may serve as an adjunctive imaging tool. Biplane ICE imaging (–45°/+ 45° view, with left flexion and counterclockwise rotation) can supply adequate images to guide grasping (Fig. 9) [25].

**Fig. 10:**
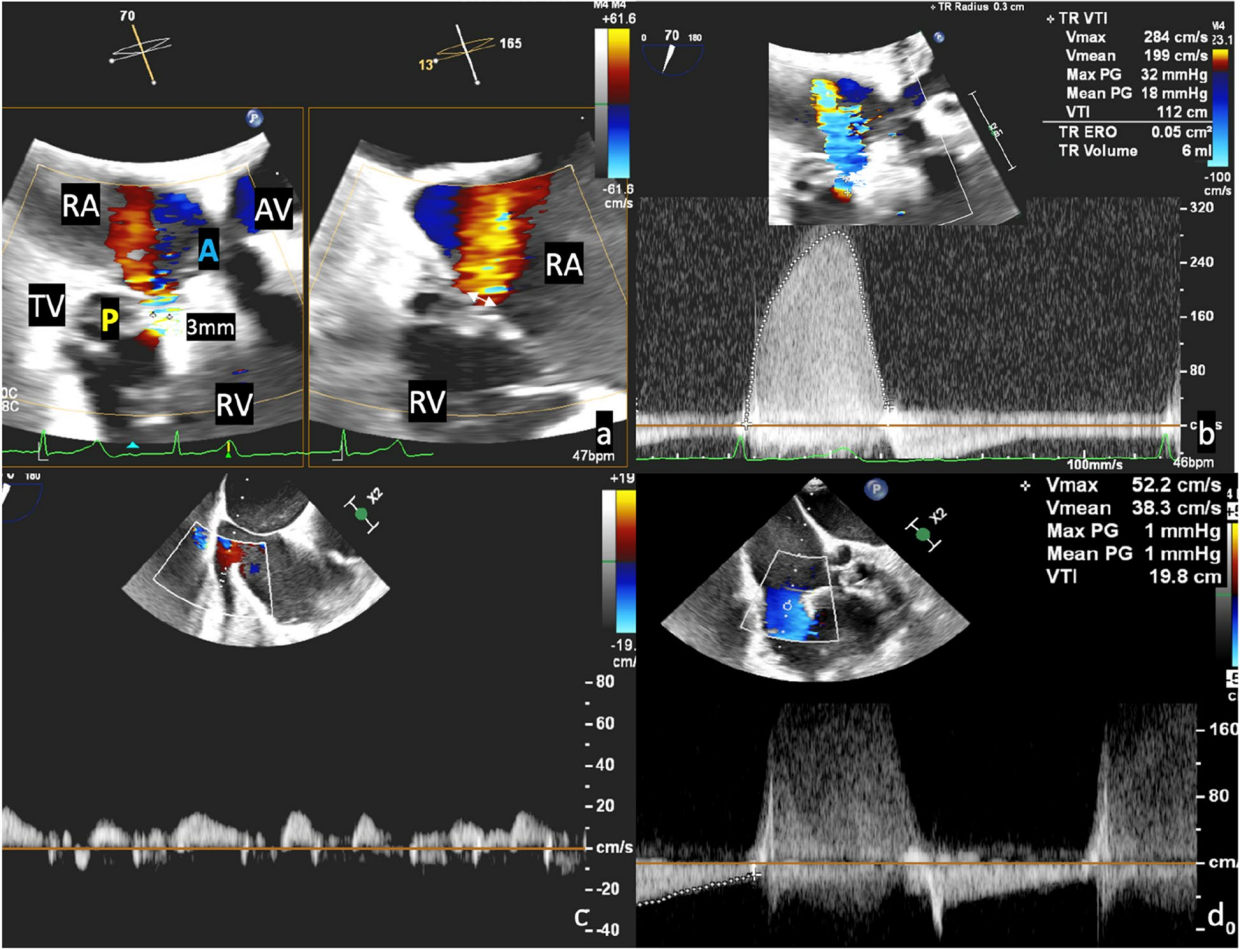
**A** The vena contracta diameter of post-clipping tricuspid regurgitation (TR), effective regurgitant orifice (ERO) area and (**B**) TR regurgitant volume, (**C**) hepatic vein Doppler interrogation, and (**D**) tricuspid valve (TV) mean gradient should be properly assessed after clip implantation. The final parameters of vena contracta 3 mm, ERO area 0.05 cm^2^ and regurgitant volume 6 mL, absence of systolic flow reversal at hepatic vein, and mean gradient 1 mmHg demonstrated a successful Both transcatheter tricuspid edge-to-edge repair procedure with significant TR reduction. A, anterior leaflet; AV, aortic valve; P, posterior leaflet; PG, ****; RA, right atrium; RV, right ventricle; VTI, ****

Postprocedural assessment must include evaluation of residual TR (multiparametric, including vena contracta width and effective regurgitant orifice) (Fig. 10A, B), mean transtricuspid gradient (should be ≤ 3 mmHg, which ensure there is no postprocedural tricuspid stenosis) (Fig. 10D), and hepatic vein flow. Abolishment of late-systolic flow reversal in the hepatic vein supports a meaningful reduction in TR severity (Fig. 10C).

## Background of TTVR

Certain TV anatomies are not favorable for T-TEER; these include a very dilated tricuspid annulus, wide coaptation gaps, excessive leaflet tethering and retraction, and CIED-related TR [10]. For those challenging TV anatomies, orthotopic TTVR is an attractive alternative with advantage of abolishing TR, which could be done through transjugular or transfemoral approaches.

### Intraprocedural guidance of LuX-Valve Plus with echocardiography

**Fig. 11:**
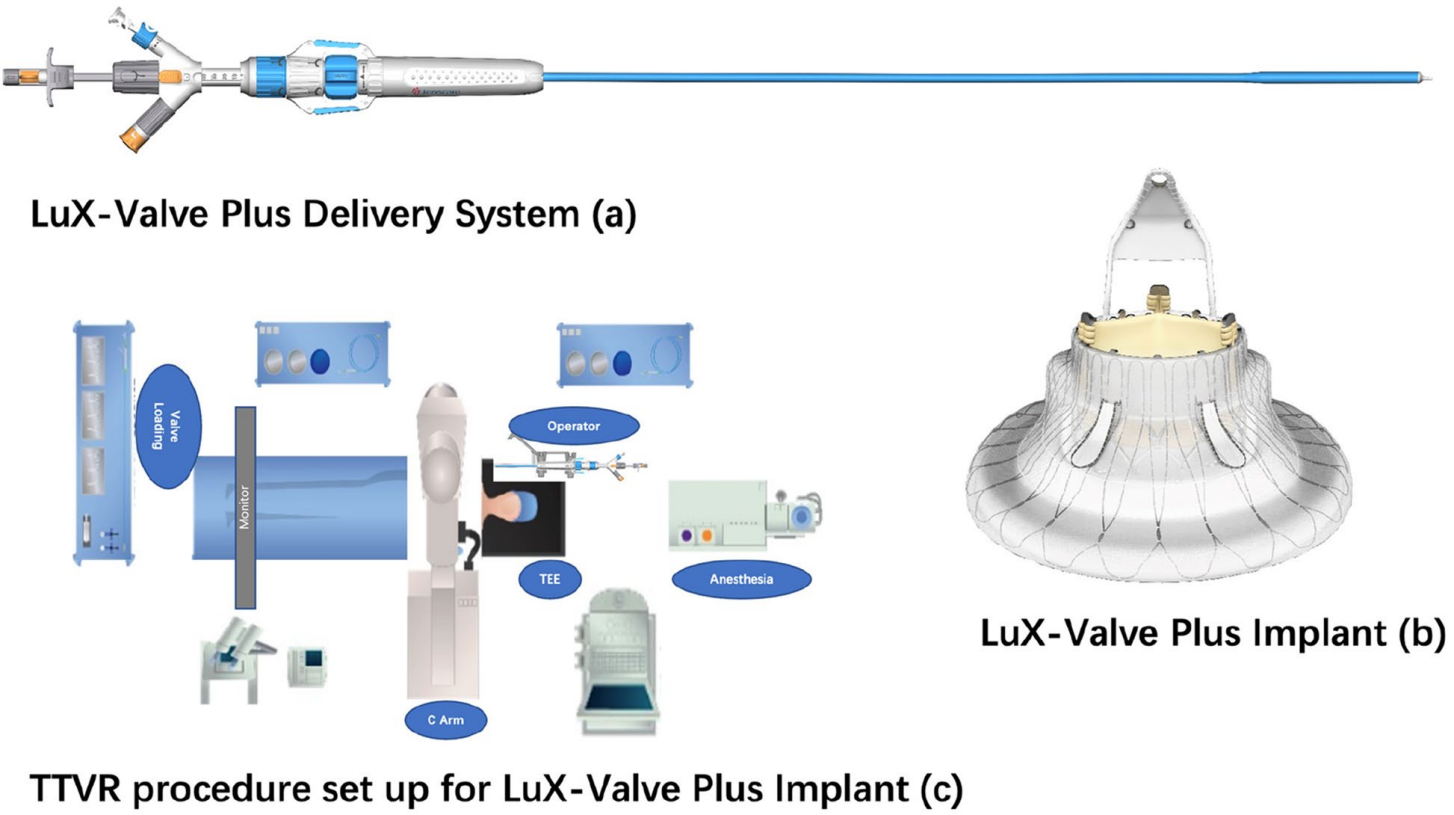
The LuX-Valve Plus (Ningbo Jenscare Biotechnology) is a novel radial force–independent orthotopic transcatheter tricuspid valve replacement (TTVR) with (**A**) the component of delivery system and (**B**) LuX-Valve Plus implant. **C** The LuX-Valve Plus is implanted through single jugular venous access under transesophageal echocardiography and fluoroscopy guidance

The LuX-Valve Plus is a novel radial force–independent orthotopic TTVR (Fig. 11A, B), with sizing ranging from 40 to 70 mm, that has proven its efficacy in substantial TR reduction, with significant right heart reverse remodeling, and improved functional status demonstrated in the single-arm, multicenter TRAVEL study [22]. Cardiac computed tomography is pivotal in assessment of tricuspid annular size and geometry, RA and RV volumes, and measurement of angle between the interventricular septum and tricuspid annulus, prior to TTVR.

**Fig. 12:**
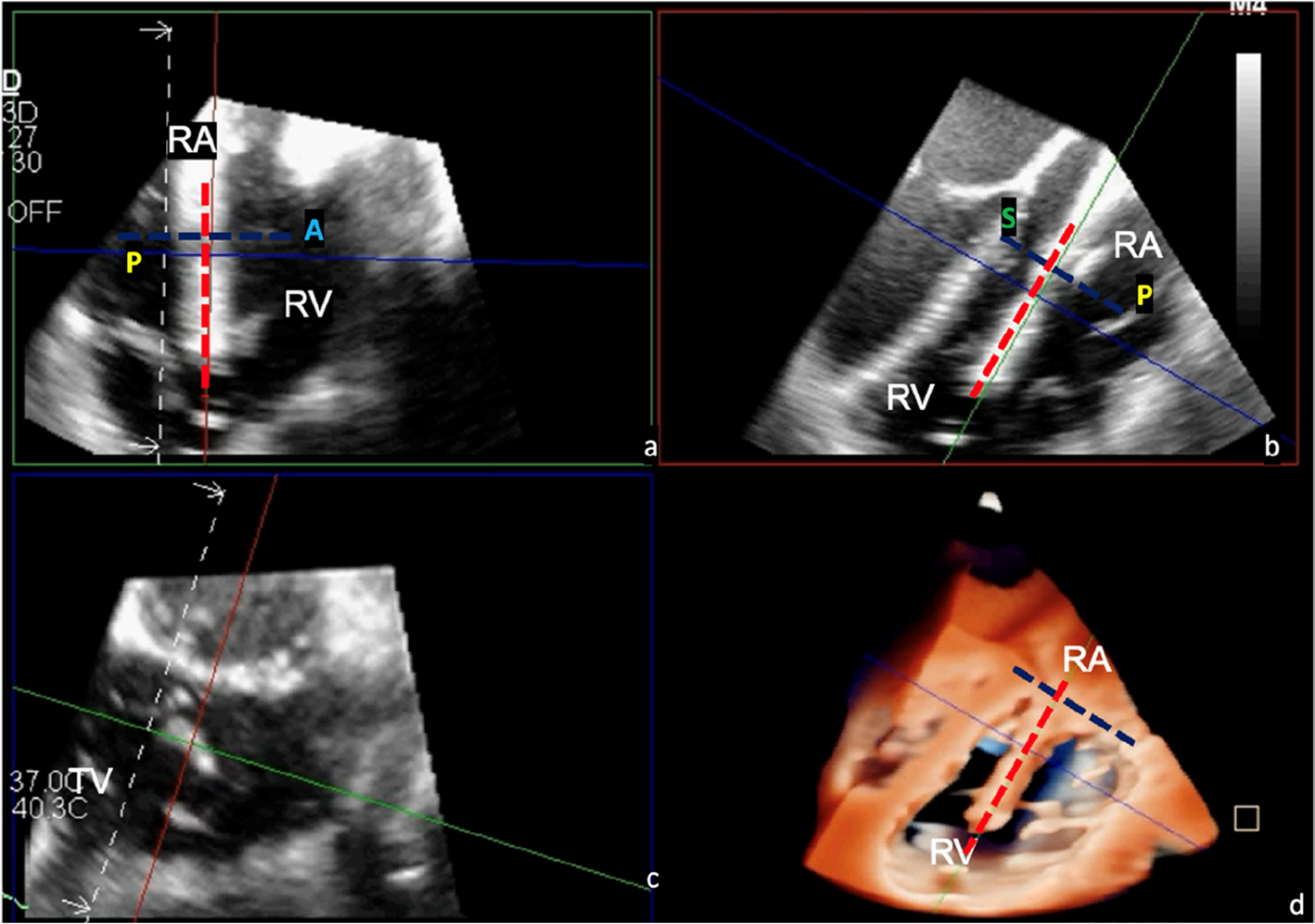
Having successful access into right ventricle, 3D multiplanar reconstruction of **A** mid-esophageal view (50°–70°) paired with **B**, **D** orthogonal four-chamber view (in 3D photorealistic imaging) provide essential images to ensure the LuX delivery system maintain appropriate co-axiality (red dotted line) and **C** central crossing through the tricuspid annulus (blue dotted line). A, anterior leaflet; P, posterior leaflet; RA, right atrium; RV, right ventricle; S, septal leaflet; TV, tricuspid valve

The LuX-Valve Plus is implanted via single jugular venous access under TEE and fluoroscopy guidance (Fig. 11C) [26]. Having established the correct internal jugular access, the valve delivery system is advanced to the mid-RA. Subsequently, the whole system is steered into the RV by flexion. Having a successful entry into the right ventricle, the 3D MPR of the ME view provides essential images to ensure appropriate co-axiality of the LuX delivery system and central crossing through the tricuspid annulus (Fig. 12). Fine-tuning is achieved by flexing and advancing for anterior adjustment, unflexing and withdrawing for posterior, counterclockwise rotation to bias the septal aspect, and clockwise rotation to bias the lateral aspect.

**Fig. 13:**
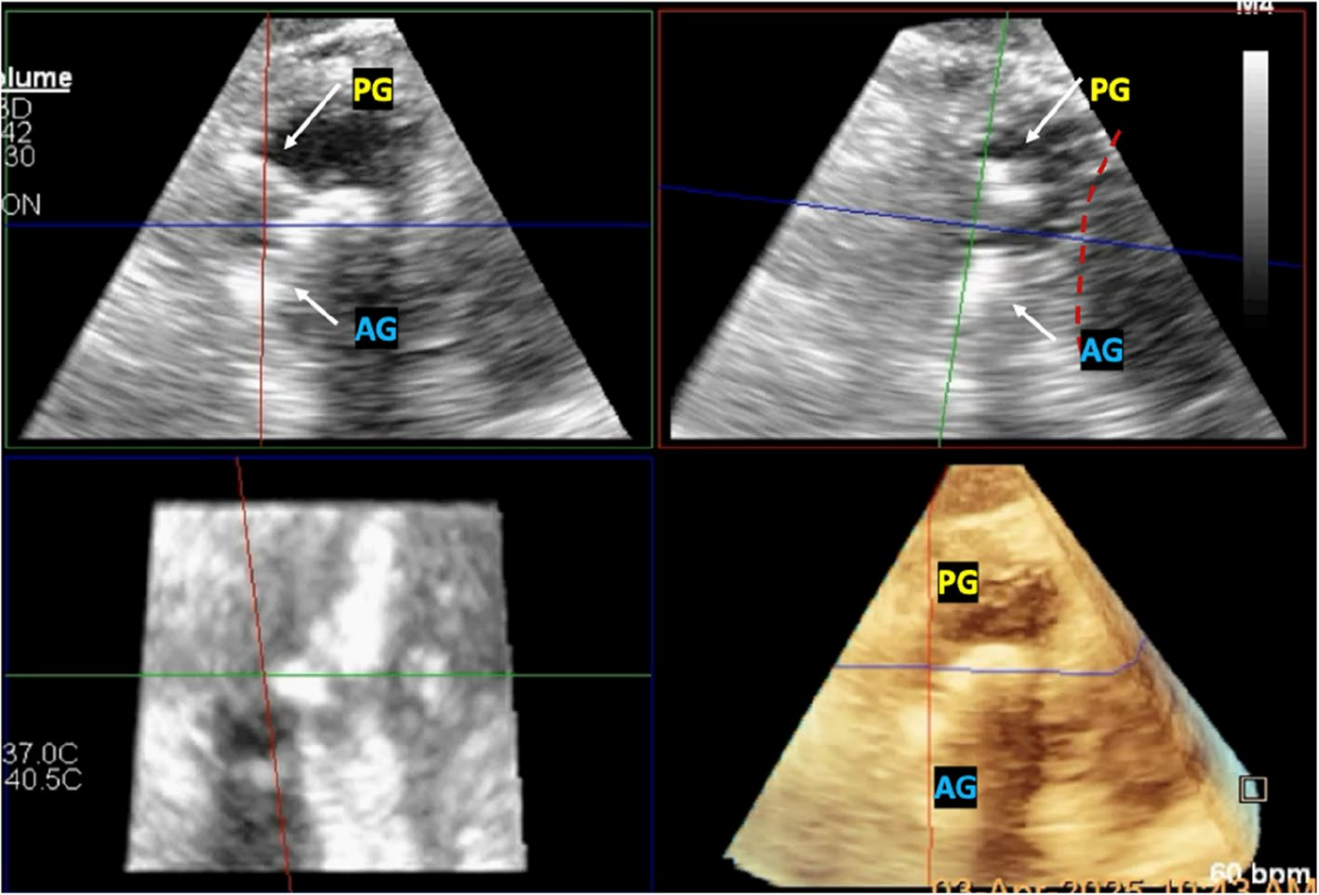
The 3D multiplanar reconstruction of transgastric view focusing on the short-axis of tricuspid valve at 0°–30° play a vital role helping to identify the anterior (AG) and posterior (PG) graspers which are just beneath the tricuspid valve annulus (red dotted line; with embedded video)

**Fig. 14:**
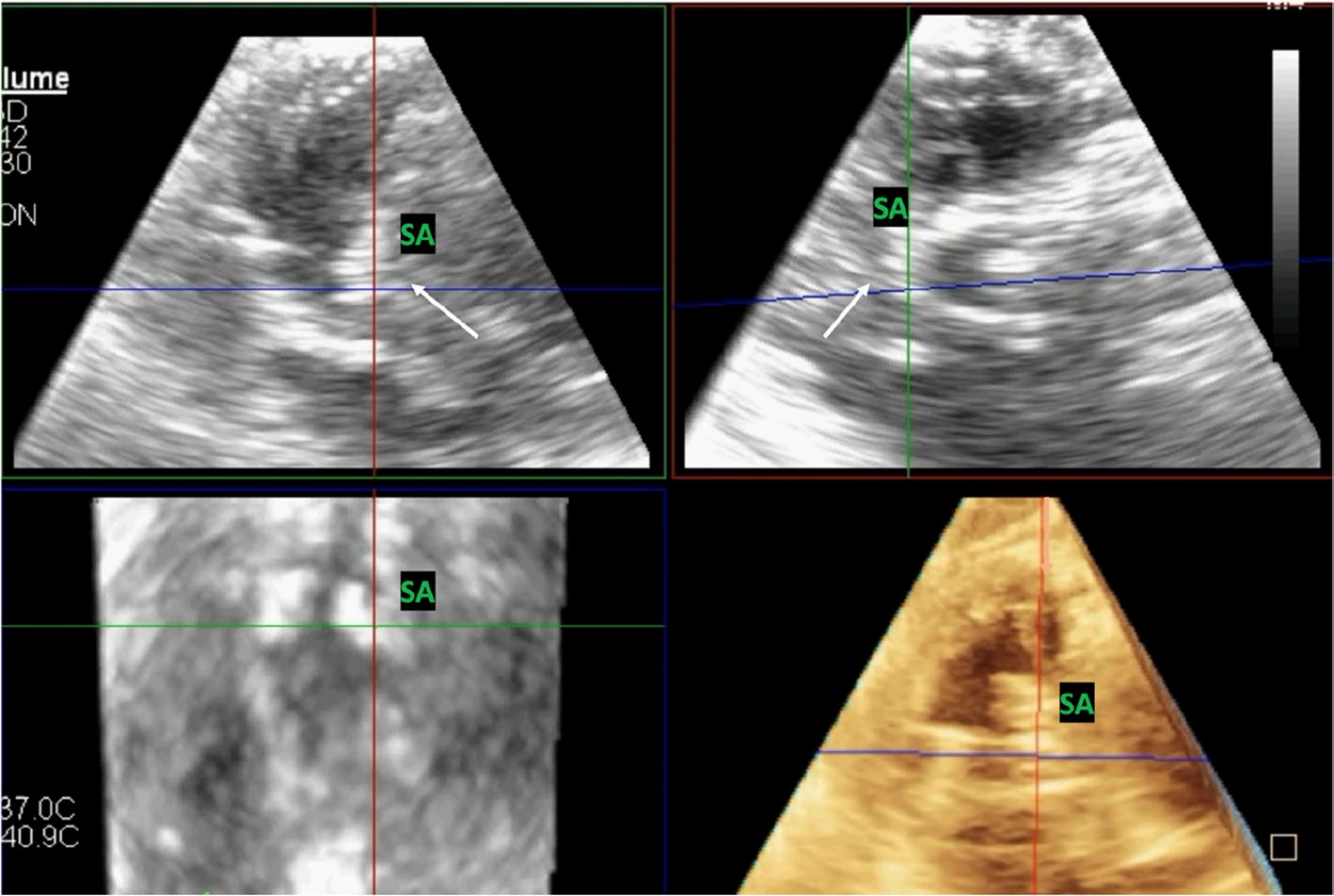
The 3D multiplanar reconstruction transgastric view on the tricuspid valve level (0°–30°) allows visualization of septal anchor (SA) tube is in optimal contact to the ventricular septum (arrow), subsequently the three hooks are released to secure the whole system (with embedded video)

Before releasing the graspers of the LuX-Valve Plus, the delivery system depth must be adjusted by the coupling mechanism to approximately 10 mm below the tricuspid annulus. Gradually, the graspers are fully exposed in a ME biplane view, a TG biplane view, or a 3D MPR, with TV used to confirm that the anterior and posterior graspers are below the anterior and posterior tricuspid valve leaflets, respectively (Fig. 13). Once both grasper locations are confirmed, the atrial skirt of the valve will be released by unsheathing the grasper. The ME RV inflow-outflow or TG 3D *en face* views are used to locate the paravalvular leak, enabling precise alignment of the LuX-Valve to achieve effective closure. In the scenario of multiple paravalvular leaks, valve apposition can be optimized by gentle ventricular advancement. As for septal anchoring, the TG view allows visualization of the septal anchor tube to optimal contact with the ventricular septum (Fig. 14), subsequently the hooks are released to secure the whole implant system. After achieving a satisfactory reduction in TR, the device was deployed successfully.

A structured postprocedural imaging framework for TTVR will be performed that includes transvalvular gradient should be ≤ 3 mmHg, residual or paravalvular TR should be ≤ 1 +, RV systolic function, and potential complications such as device malposition, migration, thrombosis, interference with adjacent structures, and new pericardial effusion.

### Intraprocedural guidance of EVOQUE TTVR with echocardiography

The EVOQUE TTVR system is currently the most comprehensively evaluated device and remains the only TTVR platform approved by the US Food and Drug Administration. The EVOQUE TTVR for compassionate use demonstrated durable efficacy, persistent symptomatic improvement, and lower rates of mortality and heart failure hospitalizations at a 1-year follow-up [27]. In the latest TRISCEND II randomized controlled trial, this device was superior to medical therapy in terms of symptom improvement and quality of life [7]. The system consists of a 27-mm trileaflet pericardial tissue valve mounted within a nitinol frame available in diameters of 44 to 56 mm. Similar to LuX-Valve Plus, EVOQUE valve sizing is based on computer tomography imaging of tricuspid annulus.

Intraprocedural imaging guidance combines fluoroscopy with TEE or 3D ICE, with a strong reliance on real-time 3D MPR to optimize device delivery, positioning, and trajectory. The primary objective is to obtain a 3D *en face* view of the TV, with a corresponding 2D short-axis plane that facilitates identification of the device anchors [28]. Imaging planes from ME, deep esophageal and sometimes TG views are required to visualize entire nine anchors of device.

**Fig. 15:**
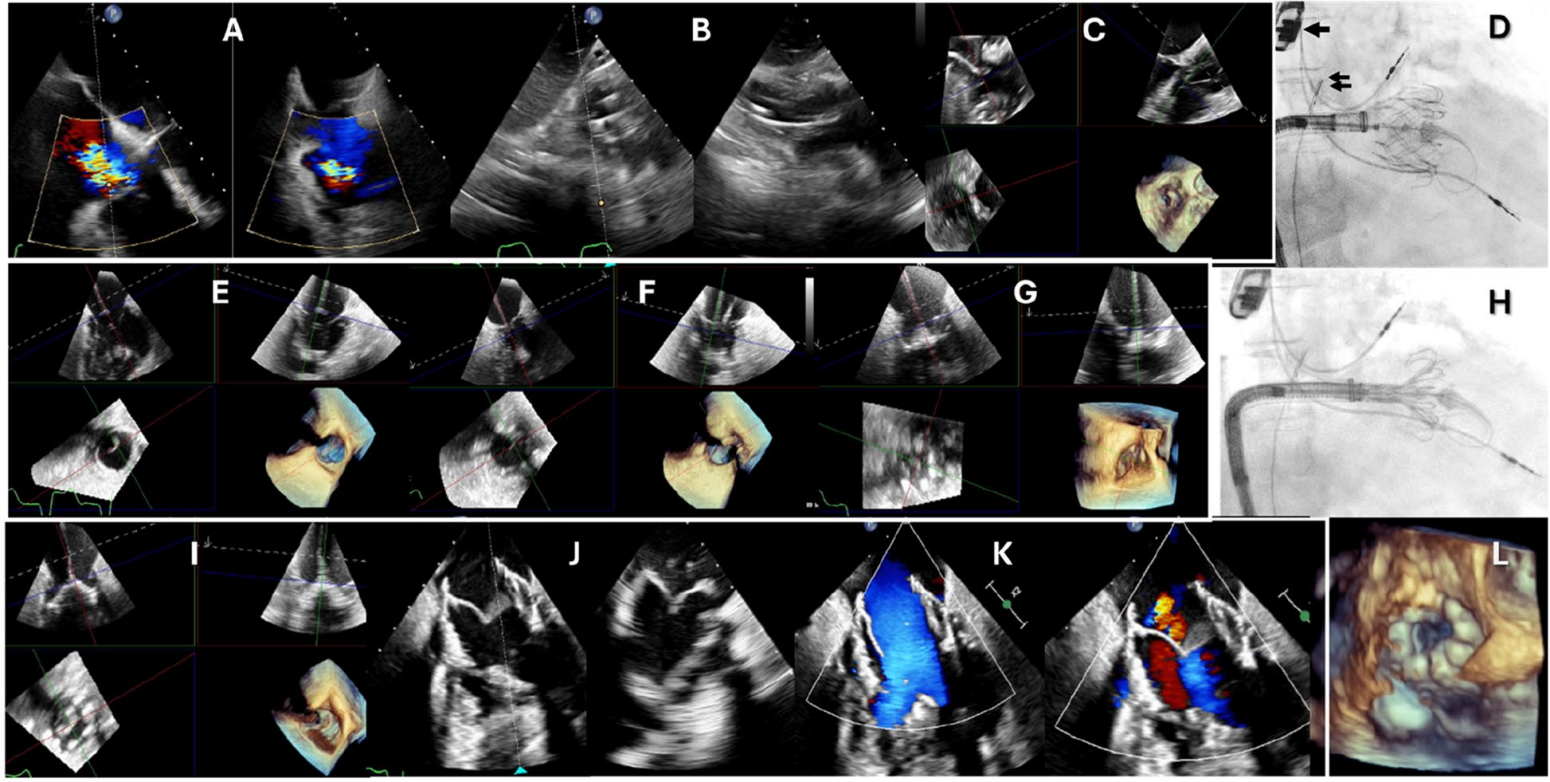
Case of transcatheter tricuspid valve replacement with the EVOQUE system (Edwards Lifesciences), for cardiac implantable electronic device–related severe tricuspid regurgitation (TR). **A** The mid-esophageal biplane views of the tricuspid valve (TV) with significant TR are shown. **B** The biplane transgastric transesophageal echocardiography (TEE) views of the TV are suboptimal to visualize the stiff wire in the right ventricle (RV); hence, **C** 3D intracardiac echocardiography (ICE; Veri Sight Pro, Philips) is used to guide the procedure. **D** The locations of the ICE catheter in the right atrium (RA; double black arrow) and the TEE probe (single black arrow) are shown in the fluoroscopic image. **E**–**G** The 3D multiplanar reconstruction (MPR)-derived from the 3D ICE images are shown with the valve delivery system entering deep in **E** the RV, **F** mid RV, and **G** close to the TV, this allows fine adjustment of device depth, position and alignment. **I** The nine anchors of the valve are exposed, and the engagement of the leaflets are shown in the coronal plane of the 3D MPR. (H) The fluoroscopic appearance of this step is shown. **J** Full ventricular expansion is followed by atrial expansion and the valve is deployed under ICE guidance (biplane ICE image of the fully deployed valve is shown). (K) ICE color flow Doppler shows unobstructed diastolic TV flow and mild transvalvular TR. (L) The *en face* view of deployed valve from RA perspective under 3D TEE

The TTVR device system is advanced through a steerable delivery catheter across the tricuspid annulus into the RV (Fig. 15C). Device position, depth, and alignment are refined using 3D MPR (Fig. 15E–G). Anchor deployment is achieved by retracting the valve delivery system, allowing placement below the tricuspid leaflets. 3D MPR enables systematic rotation of orthogonal long-axis planes centered on the short-axis view, facilitating identification of all nine anchors and confirming that the leaflets remain positioned above the anchors during systole [28].

Subsequently, the anchors are advanced beneath the annulus to achieve leaflet capture. Multiplanar rotational assessment is again required to verify engagement of all leaflets by the nine anchors (Fig. 15I). Once adequate leaflet capture is confirmed, the valve is fully expanded and detached from the delivery system (Fig. 15J). Following deployment, echocardiography guides safe withdrawal of the nosecone and is used to evaluate device stability and procedural outcomes, including residual transvalvular and paravalvular regurgitation, diastolic gradients, and overall hemodynamic improvement (Fig. 15 K, L). In addition, it is important to evaluate changes in RV size and systolic function, and the presence or evolution of pericardial effusion.

## Conclusions

TTVI, including T-TEER and TTVR for severe symptomatic TR, is gaining popularity in Asia–Pacific countries due to its minimally invasive nature. Meaningful TR reduction confers symptomatic, functional, and quality-of-life benefits. Comprehensive screening and intraprocedural TEE, supplemented by 3D-ICE, play a pivotal role in selecting appropriate anatomies and guiding a successful procedure (Table 2)**.**

**Table 2:** Standardized echocardiographic protocol for transcatheter tricuspid valve intervention

Stage	Objective	Key TEE view	T-TEER–specific checkpoint (TriClip^a)^)	TTVR-specific checkpoint (LuX-Valve Plus^b)^ and EVOQUE^c)^)	No-go/re-intervene criteria
Before procedure	Quantify TR severity (VC, EROA, etc.) Assess RV function (TAPSE, FAC) and pressure (RVSP) Identify TV coaptation gap, leaflet tethering, and morphology Demonstration of systolic flow reversal from hepatic vein	RV inflow-outflow view Four-chamber view Bicaval view TG view	TV coaptation gap assessment from TG view Anterior and posterior tricuspid leaflet lengths from RV inflow-outflow view Assessment of systolic flow reversal from hepatic vein at bicaval view Baseline mean gradient across TV	Annulus sizing confirmation Angle between tricuspid annulus and interventricular septum should be within 80°–100° (LuX-Valve Plus) Assessment of systolic flow reversal from hepatic vein Baseline mean gradient across TV	No-go: TV coaptation gap ≥ 10 mm (T-TEER) Tricuspid annulus maximum diameter ≥ 65 mm (TTVR)
During procedure	Confirm device positioning Assess leaflet capture and proper alignment	RV inflow-outflow view Four-chamber view Bicaval view TG view	Clip arm orientation is perpendicular to leaflet coaptation from TG view Bilateral leaflet insertion minimum 6 mm for NT/NTW and minimum 9 mm for XT/XTW from RV inflow-outflow view, or biplane imaging using 3D-ICE Ensure no entrapment of TV chordae or pacing/defibrillator lead	Proper positioning of anterior and posterior graspers beneath the tricuspid leaflets; septal anchor tube rotation towards the interventricular septum (LuX-Valve Plus) 3D MPR facilitates identification of all nine anchors and confirms that the leaflets are well captured by the anchors (EVOQUE) Ensure no significant paravalvular TR	No-go: Insertion < 5 mm (T-TEER) Significant device disc tilting (TTVR) Residual transvalvular or paravalvular TR ≥ 2 +
After procedure	Quantify residual TR Assess change in RV function Screen for any complications (e.g., SLDA, pericardial effusion, etc.) Device final position and stability	RV inflow-outflow view Four-chamber view Bicaval view TG view	Residual TR ≤ 2 + Mean gradient ≤ 3 mmHg across TV and abolishment of systolic flow reversal at hepatic vein Stable clip position with creation of strong tissue bridge	TR ≤ 1 + Mean gradient ≤ 3 mmHg across TV and abolishment of systolic flow reversal at hepatic vein Symmetric device alignment with and stable positioning	Re-intervene: TR ≥ 3 + TV gradient > 5 mmHg embolization/SLDA

EROA, effective regurgitant orifice area; FAC, fractional area change; ICE, intracardiac echocardiography; MPR, multiplanar reconstruction; RV, right ventricle; RVSP, right ventricular systolic pressure; SLDA, single leaflet device attachment; T-TEER, transcatheter tricuspid edge-to-edge repair; TAPSE, tricuspid annular plane systolic excursion; TEE, transesophageal echocardiography; TG, transgastric; TR, tricuspid regurgitation; TTVR, transcatheter tricuspid valve replacement; TV, tricuspid valve; VC, vena contracta

^a^^)^Abbott Laboratories.^b)^Ningbo Jenscare Biotechnology. ^c)^Edwards Lifesciences

## Data Availability

No datasets were generated or analysed during the current study.

## Abbreviations

AFTR: Atrial functional tricuspid regurgitation
CDS: Clip delivery system
CIED: Cardiac implantable electronic device
EROA: Effective regurgitant orifice area
ICE: Intracardiac echocardiography
KCCQ: Kansas City Cardiomyopathy Questionnaire
ME: Mid-esophageal
MPR: Multiplanar reconstruction
PISA: Proximal isovelocity surface area
RA: Right atrium
RV: Right ventricle
TEE: Transesophageal echocardiography
TG: Transgastric
TR: Tricuspid regurgitation
T-TEER: Transcatheter tricuspid edge-to-edge repair
TTVI: Transcatheter tricuspid valve intervention
TTVR: Transcatheter tricuspid valve replacement
TV: Tricuspid valve
VFTR: Ventricular functional tricuspid regurgitation
